# Supplementary material for: Neuropeptide signalling shapes feeding and reproductive behaviours in male Caenorhabditis elegans
Source: Life Sci Alliance. 2022 Jun 23;5(10):e202201420. doi: 10.26508/lsa.202201420 (PMC9233197; doi:10.26508/lsa.202201420)
Supplement: Supplementary file 1 [file LSA-2022-01420_TableS1.docx]

**Table S1: List of strains used in this study.** For transgenic lines, where indicated the number following each construct in brackets refers to the ng/µL of plasmid injected e.g. (50) means 50 ng/µL of the construct was included in the injection mix.

| Strain number | Genotype |
| --- | --- |
| **N2** | Wild-type |
| **AQ4214** | *fog‐2(q71)* |
| **AQ4266** | *him‐5(e1490)V bc4x* |
| **YLC022** | *him‐5(e1490) V; lury-1(gk961835)* |
| **AQ4287** | *him‐5(e1490)V; npr‐22 (ok1598) bc 5x* |
| **AQ4269** | *him‐5(e1490)V bc4x; npr‐22 (ok1598) bc 5x; lury-1(gk961835)* |
| **AQ4291** | *him‐5(e1490)V; ljEx1001[Plury-1(2kb)::lury-1::SL2‐mKate2(25);unc‐122::gfp(50)]* |
| **AQ4215** | *lury-1(gk961835);IjEx1001[Plury-1(2kb)::lury-1::SL2‐mKate2(25);unc‐122::gfp(50)]* |
| **AQ4270** | *him‐5(e1490)V bc4x; npr‐22 (ok1598) bc 5x; IjEx1001[Plury-1(2kb)::lury-1::SL2‐mKate2(25);unc‐122::gfp(50)]* |
| **AQ4290** | *him-5(e1490)V; bc4x; lury-1(gk961835) backcrossed 6x; ljEx1001[Plury-1(2kb)::lury-1::SL2-mKate2(25);unc-122::gfp(50)]* |
| **YLC103** | *him‐5(e1490); pepEx009[Plury-1(3.4kb)::lury-1 gDNA + UTR::SL2-mKate2(pYLC016)(50); unc-122::gfp (60)]- line 1* |
| **YLC104** | *him‐5(e1490); npr‐22 (ok1598); pepEx009[Plury-1(3.4kb)::lury-1 gDNA + UTR::SL2-mKate2(pYLC016)(50); unc-122::gfp (60)]- line 1* |
| **YLC105** | *him‐5(e1490); lury-1(gk961835); pepEx009[Plury-1(3.4kb)::lury-1 gDNA + UTR::SL2-mKate2(pYLC016)(50); unc-122::gfp (60)]- line 1* |
| **YLC124** | *him-5(e1490); pepEx011[Plury-1(3.4kb)::ced-3 (p15)::nz::unc-54 3’UTR; Plury-1(3.4)::cz::ced-3 (p17)::unc-54 3’UTR; Punc-122::gfp::unc-54 3’UTR] line 1* |
| **YLC125** | *him-5(e1490);pepEx012[Plury-1(3.4kb)::ced-3 (p15)::nz::unc-54 3’UTR; Plury-1(3.4)::cz::ced-3 (p17)::unc-54 3’UTR; Punc-122::gfp::unc-54 3’UTR] line 2* |
| **YLC155** | *him-5;pepEx013[Plury-1(3.4kb)::mKate2, unc-122::gfp]* |
| **LX2073** | *npr-22::GFP* fosmid |
| **YLC195** | *Ex[*P*lury-1(3,458bp)::venus,unc-122p::mCherry], otIs670[NeuroPAL]* |
